# Effectiveness of Implant Therapy in Patients With and Without a History of Periodontitis: A Systematic Review With Meta‐Analysis of Prospective Cohort Studies

**DOI:** 10.1111/jre.13351

**Published:** 2024-10-28

**Authors:** Marco Annunziata, Gennaro Cecoro, Agostino Guida, Gaetano Isola, Paolo Pesce, Roberto Sorrentino, Massimo del Fabbro, Luigi Guida

**Affiliations:** ^1^ Multidisciplinary Department of Medical‐Surgical and Dental Specialties University of Campania “Luigi Vanvitelli” Naples Italy; ^2^ U.O.C. Odontostomatologia A.O.R.N. “A. Cardarelli” Naples Italy; ^3^ Department of General Surgery and Surgical‐Medical Specialties, School of Dentistry University of Catania, AOU “Policlinico‐San Marco” Catania Italy; ^4^ Department of Surgical Sciences and Integrated Diagnostics (DISC) Genova University Genoa Italy; ^5^ Department of Neurosciences, Reproductive and Odontostomatological Sciences, Division of Prosthodontics, Scientific Unit of Digital Dentistry University “Federico II” of Naples Naples Italy; ^6^ Department of Biomedical, Surgical and Dental Sciences University of Milan Milan Italy; ^7^ Fondazione IRCCS Ca' Granda Ospedale Maggiore Policlinico Milan Italy

**Keywords:** dental implants, implant loss, implant survival, marginal bone loss, observational studies, peri‐implant diseases, periodontal diseases

## Abstract

**Aim:**

This systematic review investigates the effectiveness of implant therapy in patients with and without a history of periodontitis in terms of implant loss, peri‐implant marginal bone loss (MBL), and occurrence of peri‐implant diseases.

**Methods:**

The protocol of the present meta‐analysis was registered on PROSPERO (CRD42021264980). An electronic search was conducted up to April 2024. All prospective cohort studies reporting implant loss, MBL, and occurrence of peri‐implant diseases in both patients with a history of periodontitis (HP) and patients with no history of periodontitis (NHP) after at least 36‐month follow‐up were included. The risk of bias was evaluated using the Newcastle‐Ottawa Scale and the quality of the evidence was also assessed. A meta‐analysis was performed on the selected outcomes at the available follow‐up time points. Subgroup analyses were conducted based on follow‐up time, rate of progression and severity of periodontitis, and implant surface characteristics. Publication bias was evaluated using the Funnel plot and Egger's test.

**Results:**

From 13 761 initial records, 14 studies (17 articles) were finally included. Eight studies had a low risk of bias level, and six had a medium risk of bias level. Meta‐analysis showed that HP patients had a significantly greater risk for implant loss (HR: 1.75; 95% CI: 1.28–2.40; *p* = 0.0005; *I* ^2^ = 0%), MBL (MD: 0.41 mm; 95% CI 0.19, 0.63; *p* = 0.0002; *I* ^2^ = 54%), and peri‐implantitis (3.24; 95% CI: 1.58–6.64; *p* = 0.001; *I* ^2^ = 57%) compared to NHP, whereas no significant intergroup difference for peri‐implant mucositis was found. Subgroup analyses revealed a particularly greater risk for implant loss for HP patients over a ≥ 10‐year follow‐up (HR: 2.02; 95% CI: 1.06–3.85; *p* = 0.03; *I* ^2^ = 0%) and for patients with a history of grade C (formerly aggressive) periodontitis (HR: 6.16; 95% CI: 2.53–15.01; *p* < 0.0001; *I* ^2^ = 0%). A greater risk for implant loss for stages III–IV (severe) periodontitis, and implants with rough surfaces was also found.

**Conclusions:**

Within the limits of heterogeneous case definitions and methods of assessment, a history of periodontitis has been proved to significantly increase the risk for implant loss, particularly at long follow‐up (≥ 10 years) and in case of rapidly progressive forms (grade C), and for MBL and peri‐implantitis.

## Graphical abstract

A history of periodontitis significantly increases the risk for implant loss, peri‐implant marginal bone loss and peri‐implantitis.

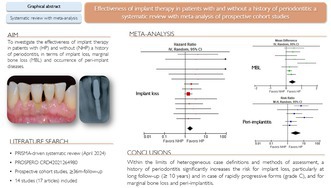

## 1 Introduction

Periodontitis is the most common chronic inflammatory noncommunicable human disease and the main cause of tooth loss in adult populations in industrialized countries [1]. In the last decades, the use of osseointegrated dental implants has become an established and widely used treatment option for rehabilitating both fully and partially edentulous patients [2, 3]. The high prevalence of peri‐implant diseases, however, between 43% and 46.83% for peri‐implant mucositis and between 19.5% and 22% for peri‐implantitis at patient level [4, 5, 6, 7], has gained attention in recent years, consistently with the increased use of dental implants for oral rehabilitations. Following the principles of primordial prevention of peri‐implant diseases, in patients awaiting implant placement, complete patient information, thorough assessment of the patient's risk profile to identify and manage modifiable risk factors/indicators for peri‐implant diseases, as well as guideline‐conformed treatment of periodontal disease to a stable endpoint and adherence to a supportive care program prior to implant placement are strongly recommended [6]. Nevertheless, even in the case of a completely stable periodontal condition (i.e., periodontal health on a reduced periodontium in a patient with a history of periodontitis), such a patient still remains at increased risk of recurrent progression of periodontitis [8] and a history of periodontitis has been indicated as a risk indicator of peri‐implantitis together with poor plaque control skills and no regular maintenance care after implant therapy [9, 10, 11]. In fact, a double link between periodontitis and implant treatment exists: periodontitis is the main cause of tooth loss in adults, so a large part of patients requiring implant treatment have a history of periodontitis and, in turn, implant treatment, and its prognosis in the long term, may significantly be affected by a history of periodontitis.

Although several longitudinal studies have shown evidence of a negative impact of the history of periodontitis on implant prognosis, systematic reviews on this topic have failed to draw definitive conclusions in the past [12, 13]. In many cases, the high heterogeneity of the included studies did not allow for quantifying the risk by reliable meta‐analyses of available data. Some authors have reported a significantly greater risk of implant loss in patients with a history of periodontitis than in patients with no history of periodontitis, with a risk ratio (RR) ranging from 0.25 to 1.89 [14, 15, 16], whereas some other studies reported no significant difference in the risk for implant loss between patients with and without a history of periodontitis [17, 18].

More recent meta‐analyses supported the association between a history of periodontitis and implant loss, including studies with variable design [19] or focusing exclusively on prospective cohort studies [20] to limit the bias due to retrospective and cross‐sectional studies and strengthen the evidence. Both these studies have investigated the role of the observation time (follow‐up) for this association to become evident, but no univocal conclusions could be reached. While, indeed, Carra et al. [19] affirmed that follow‐ups shorter than 5 years appear to be insufficient to detect a significant difference in implant survival between patients with and without a history of periodontitis, Serroni et al. [20] found a significant effect also for studies with a ≤ 5‐year follow‐up. Other variables possibly affecting the true relationship between exposure and outcome, including the characteristics of implants (surface modifications) and of periodontitis patients experienced (severity and rate of progression), were not assessed. Furthermore, other outcomes, such as the occurrence peri‐implant mucositis, were not examined by previous similar studies.

In virtue of these considerations, we aimed, with this study, to evaluate the effectiveness of implant therapy in patients with and without a history of periodontitis in terms of implant loss, marginal bone loss (MBL), and occurrence of peri‐implant diseases through the systematic assessment of the available scientific evidence exclusively derived from prospective studies, evaluated for study bias by the Newcastle‐Ottawa Scale (NOS), meta‐analyzed with sensitivity and subgroup analyses for relevant variables, and assessed for quality of evidence.

## 2 Methods

The 2020 version of the Preferred Reporting Items for Systematic Reviews and Meta‐Analyses (PRISMA) statement [21] was followed. The review protocol was registered in the International Prospective Register of Systematic Reviews (PROSPERO: CRD42021264980). The following focused question, according to the PECO(ST) framework [22], aimed to be answered: what is the effectiveness of implant therapy in adult patients with missing teeth (population), with a history of periodontitis (exposure), compared to patients without a history of periodontitis (comparison), in terms of risk of dental implant loss (primary outcome), peri‐implant MBL, peri‐implant mucositis and peri‐implantitis (secondary outcomes), basing on prospective cohort studies (study type) with a minimum follow‐up of 36 months (time).

### 2.1 Search Strategy and Data Sources

A computerized, systematic search of the literature was performed using MEDLINE (PubMed, www.ncbi.nlm.nih.gov/pubmed), clinicaltrials.gov, and Embase. The detailed search strategy for each electronic database consulted is presented in Table S1. The last electronic search was performed on April 2024. In addition, a manual search was performed, including gray literature, field experts, printed scientific journals, and reference lists of previous systematic reviews.

### 2.2 Selection Criteria

Exclusively prospective cohort studies comparing patients with a history of periodontitis (HP) and patients with no history of periodontitis (NHP) submitted to dental implant therapy that reported data on implant survival/loss, and/or peri‐implant bone level changes and/or occurrence of peri‐implantitis or peri‐implant mucositis, with a follow‐up ≥ 36 months were included. Conference abstracts or full‐text studies with unclear designs, unclear definitions of the study groups, or incomplete data were excluded.

### 2.3 Study Selection and Data Extraction

Two independent reviewers (MA and GC) initially screened all articles based on titles and abstracts and imported them to a reference manager to remove duplicates. Afterward, full texts were carefully examined and included or excluded by selection criteria. Any disagreement was resolved via discussion with a third author (MDF). Agreement between reviewers was assessed using Cohen's kappa coefficient (*k*). Data concerning patient and treatment characteristics and clinical outcomes for each available follow‐up for primary and secondary outcomes were independently extracted from all eligible studies by the same two reviewers. A third independent reviewer (MDF) verified the correctness of the extracted data from each primary study. When more articles referred to the same study, data were compared and, if possible, integrated. When additional information was required, authors were contacted. Two patient groups were created: patients with a history of periodontitis and patients with no history of periodontitis. The latter term was preferred to “periodontally healthy patients,” due to the lack, in most of the literature before the 2018 classification of periodontal disease, of a specific case definition of “periodontal health,” and the possible inclusion of patients with gingivitis. Implant loss was considered as the primary outcome. It was defined as the ratio between the number of implants lost at the follow‐up and those originally placed (implant level). The following secondary outcomes, both at patient and implant levels, were considered: MBL, defined as the difference in crestal bone height between baseline (implant placement or implant loading) and follow‐up measures; peri‐implantitis and peri‐implant mucositis rate, as the number of patient/implants that experienced peri‐implantitis or peri‐implant mucositis over the total number of patient/implants throughout the follow‐up.

For each study, the following data were extracted and recorded, when available, using a dedicated form to facilitate the process: first author name, year of publication, study country, study setting (private practice or university), mean duration of follow‐up, implant brand, implant surface, total number of patients and implants for each group, average patients age, patients gender, systematic diseases associated with the mechanisms of osseointegration (e.g., diabetes mellitus, osteoporosis), smoking patients, insertion of postextractive implants, bone augmentation procedures performance, type of prosthesis, implant location, supportive periodontal care (SPC) and Supportive Peri‐Implant Care (SPIC) timing, funding sources, implant survival (or failure) rate, reasons for implant failure, periodontitis classification, peri‐implantitis and peri‐implant mucositis definition, incidence of peri‐implantitis and peri‐implant mucositis, as well as radiographic peri‐implant parameters (MBL), with their definition and assessment parameters.

### 2.4 Risk of Bias Assessment

The risk of bias was independently assessed by two authors (AG and RS) according to the NOS [23], a tool for evaluating the methodological quality of meta‐analyses of observational studies. Any discrepancies between the two investigators were resolved through consensus, with a third investigator (LG) consulted in case of uncertainty.

### 2.5 Quality of Evidence

The quality of evidence emerging from the analysis of primary and secondary outcomes was assessed by applying criteria in concordance with previously published reviews [13, 24]. Associations with significant random‐effect sizes (i.e., *p* < 0.05) were graded as convincing (Class I), highly suggestive (Class II), suggestive (Class III), or weak (Class IV) evidence (Table S2).

### 2.6 Data Analysis

A meta‐analysis was performed on primary and secondary outcomes. When more than one article referred to the same patient cohort, the one with the longer follow‐up was considered. Negative and positive MBL values were used to indicate bone loss and bone gain, respectively. Effect sizes were displayed as mean difference (MD) for continuous variables or Mantel–Haenszel (MH)‐weighted risk ratio (RR) for dichotomous variables, respectively, with 95% confidence intervals (CIs). Hazard ratio (HR) and 95% CI, to compare implant loss between patient groups, were calculated as previously described [25]. Forest plots were created to illustrate the effects of the different studies and global effect estimation. RevMan 5 software (Review Manager, version 5.4, the Cochrane Collaboration, 2020, London, UK) was used to perform the statistical analyses. Statistical significance was defined as a *p* < 0.05. Heterogeneity was assessed by the *I* ^2^ statistic, and values of 25%, 50%, and 75% were considered as low, moderate, and high heterogeneity, respectively. Random‐effect models were used to adopt a more conservative approach, as a significant interstudy heterogeneity was expected [26]. Funnel plots were considered a tool for assessing publication bias if the meta‐analysis contained enough trials to make a visual inspection meaningful (10 trials minimum). Quantitative evaluation was obtained by Egger's test. The robustness of the results and the potential sources of heterogeneity were explored by performing subgroup and sensitivity analyses whenever indicated.

## 3 Results

The electronic search retrieved a total of 13 761 items. After duplicate removal, 12 577 articles were screened and 38 remained after the title and abstract evaluation. The full‐text assessment led to the exclusion of other 21 articles (Table S3). Seven authors were contacted and asked to provide missing data; one responded (14%). The *k* value for the inter‐reviewer agreement for potentially pertinent papers was 0.865 (for selecting titles and abstracts) and 0.894 (for selecting full‐text articles). Finally, data from 17 articles published between 2003 and 2023 [27, 28, 29, 30, 31, 32, 33, 34, 35, 36, 37, 38, 39, 40, 41, 42, 43] derived from 14 studies (one study participated with two reports and another study with three reports) were included. The selection process is shown in Figure S1.

### 3.1 Characteristics of the Included Studies

The characteristics of the 14 included studies (17 articles) [27, 28, 29, 30, 31, 32, 33, 34, 35, 36, 37, 38, 39, 40, 41, 42, 43] are summarized in Tables 1, 2, 3.

**TABLE 1:** Characteristics of the included studies.

Author year Reference Country—Settings	F‐U	Imp. Brand—Surface	#NHP/#imp.	#HP/#imp.	Age, mean or range—Gender (M/F)	Comorb.	Smoking pt (#smokers/ #nonsmokers)	Bone aug./post‐ex imp.	Prosthesis—Location	SPC/SPIC timing	Funding sources
Karoussis et al. 2003 [31] Switzerland—Univ.	10 y	Straumann—TPS	45/91	8/21	NR (NR)	NR	12/41	No	FPD—NR	3–6 m	Clinical Research Foundation for the Promotion of Oral Health, University of Berne; Papavramides Foundation, Universities of Athens. Senior author recipient of ITI Fellowship for 2001/2002
Mengel & Flores‐de Jacoby 2005 [34] Germany—Univ.	3 y	Nobel; Biomet 3i—Hybrid; Machined	12/30	AP 15/77 CP 12/43	19–59 (NHP 7/5, HP: CP 6/6, AP 8/7)	None	Excluded	No	FPD—Max and Mand	3 m	NR
Mengel & Flores‐de‐Jacoby 2005 [35] Germany—Univ.	3 y	Nobel—Machined	10/11	10/15	24–45 (NHP 2/8, HP 0/10)	None	Excluded	Yes (bone aug.)	FPD—Max	3 m	NR
Mengel et al. 2007 [36] Germany—Univ.	3 y	Biomet 3i—Hybrid	8/13	9/41	NHP 34 (4/4) HP 31 (5/4)	None	Excluded	No	FPD, OD—Max, and Mand	3 m	NR
Mengel et al. 2007 [33] Germany—Univ.	10 y	Nobel—Machined	5/7	5/36	NHP 31–44 (2/3) HP 20–51 (0/5)	One pt developed multiple sclerosis	NR	No	FPD, OD—Max and Mand	3 m	NR
Gatti et al. 2008 [30] Italy—Priv.	5 y	Nobel, Zimmer, Mathys, Strauman, Dentsply—Machined and Rough	29/72	MP 7/26 SP 26/129	NHP 40 (9/20) HP: MP 56 (6/1), SP 56 (10/6)	None	14/48 H 6/29 MP 3/26 SP 5/26	Yes (bone aug.)	FPD, OD—Max, and Mand	3–4–6 m	NR
De Boever et al. 2009 [28] Belgium—Univ.	4 y (mean)	Straumann—SLA and TPS	110/261	CP 68/193 AP 16/59	53.8 (47.4%/52.6%)	Included	194/513; H 110/261 CP 68/193 AP 16/59	Yes (bone aug.)	FPD—Max and Mand	6 m	Partially supported by ITI, Switzerland
Roccuzzo et al. 2010, 2012 [39, 41] Italy—Priv.	10 y	Straumann—TPS	28/61	MP 36/95 SP 37/90	NHP 45 (NR) HP: MP 49 (NR), SP 44 (NR)	None	H 28/61 MP 37/95 SP 36/90	No	FPD—NR	Ind. tail	NR
Levin et al. 2011 [32] Israel—Priv.	4.5 y (mean)	NR	283/747	MP 149/447 SP 285/1065	51.13 (NHP 111\172, HP: MP 50\99, SP 112\173)	Included	H 32/251 MP 16/133 SP 55/230	No	NR—Max and Mand	6 m	Self‐funded
Swierkot et al. 2012 [43] Germany—Univ.	8.25 y (mean)	Nobel; Biomet 3i—Machined; Hybrid	18/30	35/149	27–56 (NHP 18/30, HP 35/149)	None	14/39	Yes (bone aug.)	FPD, OD—Max, and Mand	3 m	NR
Roccuzzo et al. 2014, 2022, 2023 [37, 38, 40] Italy—Priv.	10 y, 20 y	Straumann—SLA	32/54	MP 46/96 SP 45/102	NHP 43.3 (NR) HP: MP 53.3 (NR), SP 52.7 (NR)	Uncontrolled metabolic diseases excluded	H 5/27 MP 6/40 SP 10/35	No	FPD—NR	Ind. tail	NR
Degidi et al. 2016 [29] Italy—Priv.	10 y	Dentsply—Etched sand‐blasted	82/203	32/81	53.1 (NR)	None	34/80	Yes (post‐ex.)	FPD—Max and Mand	6 m	NR
Roccuzzo et al. 2017 [42] Italy—Priv.	10 y	Straumann—SLA	23/43	18/39	48.5 (13/28)	None	NR	No	FPD—NR	Ind. tail	NR
Akram et al. 2019 [27] Saudi Arabia—Univ.	3 y	Straumann—SLA	7/11	7/48	HP 25–43 (2/5) NHP 36–47 (0/7)	None	NR	NR	FPD—Max and Mand	3 m	Deanship of Scientific Research, King Saud University, Grant/Award Number: RGP‐1438‐024

Abbreviations: AP, aggressive periodontitis; aug., augmentation; Comorb., comorbidities: CP, chronic periodontitis; FPD, fixed partial denture; F‐U, Follow‐up; HP, history of periodontitis; Imp., implants; Ind. tail., individually tailored; M/F, male/female; m, months; Mand.;, mandible; Max., maxilla; MP, moderate periodontitis; NHP, no history of periodontitis; NR, not reported; OD, overdenture; post‐ex, post‐extractive; Priv., private practice; pt, patients; SLA, sandblasted, large grit, acid‐etched; SP, severe periodontitis; SPC/SPIC, supportive periodontal/peri‐implant care; TPS, titanium plasma sprayed; Univ., university; y, years.

### 3.2 Number of Studies, Reports, Patients, and Implants

Two articles report data from the same cohort at different follow‐up times [37, 38], while an adjunctive article separately reports data regarding peri‐implant diseases [40]. Two articles report data from the same cohort at the same follow‐up: one paper reports implant loss and radiographic bone loss, while other clinical data are presented in a separate paper [39, 41]. The total number of patients analyzed in the studies included in the present systematic review was 1612, 916 HP and 696 NHP patients. A total of 4389 implants were analyzed, 2780 in HP patients and 1609 in NHP patients. The age of enrolled patients ranged from 19 to 59 years.

### 3.3 Case Definition of Periodontitis

Case definitions of periodontitis varied among the included studies. Following the current classification of periodontal and peri‐implant diseases and conditions [44], a patient is a periodontitis case in the context of clinical care if interdental CAL is detectable at ≥ 2 nonadjacent teeth, or buccal or oral CAL ≥ 3 mm with pocketing > 3 mm is detectable at ≥ 2 teeth. None of the included studies refer to this case definition of periodontitis, since they all started before the publication of the current classification in 2018, nor do they refer *ex‐post* to this case definition. Nevertheless, it is assumable that all the patients reported in these studies as HP fall under this definition.

Two studies [29, 42] did not give details about the diagnostic criteria of periodontitis adopted. In particular, Degidi, Nardi, and Piattelli [29] reported to include patients previously treated for periodontitis, referring to the study of Sbordone et al. [45], where the authors include patients treated for adult periodontitis following the 1989 AAP Classification [46]. Roccuzzo et al. [42] divide their cohort of patients into periodontally compromised patients and nonperiodontal patients without reporting or referring to any case definition.

Four studies (seven articles) included patients with chronic periodontitis [31, 32] (or excluded patients with aggressive periodontitis [37, 38, 39, 40, 41]), five studies [27, 33, 35, 36, 43] included patients with aggressive periodontitis, two studies [28, 34] included both chronic and aggressive periodontitis patients. Furthermore, four studies (seven articles) [30, 32, 37, 38, 39, 40, 41] differentiated between moderate and severe periodontitis based on AAP 1999 (Armitage's classification) criteria [27, 28, 32, 33, 34, 35, 36, 43]; Periodontal Screening and Recording (PSR) [30], based on bleeding on probing (BOP), calculus accumulation, and probing depth (PD); and a score (“S score”) determined by the number and depth of periodontal pockets [37, 38, 39, 40, 41].

### 3.4 Other Study and Population Characteristics

All the patients in the included studies received periodontal therapy before implant installation and were inserted into an SPC/SPIC program. The time interval for SPC/SPIC recall sessions ranged from 3 to 6 months in the different studies or were individually tailored [37, 38, 39, 40, 41]. One study did not highlight significant differences in smokers versus nonsmokers in terms of implant survival [31], while Degidi, Nardi, and Piattelli reported a significantly greater implant failure rate for smoker patients [29]. All the studies excluded patients with uncontrolled systemic diseases that could affect dental implant therapy outcomes. Only two studies [28, 32] accounted for patient's systemic conditions in the analysis of the results. Levin et al. [32] did not find an effect of diabetes status on the outcome analyzed, while De Boever et al. [28] found that impaired systemic health reduced implant survival in aggressive periodontitis patients. Most of the studies loaded implants in a time range from 1.5 to 6 months. One study also performed immediate loading [30], while another exclusively evaluated immediately restored implants [29].

### 3.5 Outcomes Analyzed and Case Definitions of Peri‐Implant Diseases

The outcomes of the included studies are summarized in Table 2. Implant survival or loss was reported in all the included studies. The methods used to assess radiographic MBL varied among the selected studies (Table 3).

**TABLE 2:** Outcomes of the included studies: Implant loss, peri‐implant marginal bone loss, peri‐implantitis rate, peri‐implant mucositis rate; reasons for implant loss.

Study	Imp. loss (imp. level)	Reason for imp. loss	Peri‐implant marginal BL	Peri‐implantitis rate	Peri‐implant mucositis
Karoussis et al. (2003) [31]	HP 2/21 NHP 3/91	NR	HP: mesial: 1.00 (±1.38) mm; distal: 0.94 (±0.73) mm NHP: mesial: 0.48 (±1.10) mm; distal: 0.50 (±1.08) mm	HP: 8/21 imp. NHP: 5/91 imp.	NR
Mengel and Flores‐de Jacoby (2005) [34]	HP 2/120 NHP 0/30	Mobility	HP: CP: 1 y: 0.68 (±0.54) mm; 3 y: 0.18 (±0.11) mm; AP: 1 y: 0.83 (±0.71) mm; 3 y: 0.31 (±0.22) mm NHP: 1 y: 0.58 (±0.45) mm; 3 y: 0.12 (±0.08) mm	NR	NR
Mengel and Flores‐de‐Jacoby (2005) [35]	HP 0/11 NHP 0/15	None	HP: 1 y: 1.17 mm; 3 y: 1.78 mm NHP: 1 y: 1.13 mm; 3 y: 1.40 mm	NR	NR
Mengel et al. (2007) [36]	HP 1/41 NHP 0/13	Lack of osseointegration	HP: 1 y: 1.02 (±0.89) mm; 3 y: 1.29 mm NHP: 1 y: 0.52 (±0.23) mm; 0.71 mm	NR	NR
Mengel et al. (2007) [33]	HP 3/36 NHP 0/7	Mobility	HP: 2.07 mm 1 y after loading, 1.3 subsequent 9 y NHP: 1.13 mm 1 y after loading, 0.11 subsequent 9 y	NR	NR
Gatti et al. (2008) [30]	HP: MP: 0/26; SP: 2/129 NHP: 0/72	Peri‐implantitis	HP: MP: 2.80 (±0.45) mm; SP: 2.63 (±1.06) mm NHP: 1.37 (±1.04) mm	HP: MP: 0/36 imp., 0/7 pt SP: 4/129 imp., 2/26 pt NHP: 0/72 imp., 0/29 pt	NR
De Boever et al. (2009) [28]	HP: CP: 7/193; AP: 9/59 NHP: 8/261	NR	HP: CP: 79% mesial and 76% distal sides no BL; AP: 68% mesial and 74% distal sides no BL. NHP: 87.8% imp. no BL.	Signs of inflammation and peri‐implantitis in 12.7% imp.	NR
Roccuzzo et al. (2010, 2012) [39, 41]	HP: MP: 7/95; SP: 9/90 NHP: 2/61	Peri‐implantitis	HP: MP: 1.14 (±1.11) mm; SP: 0.98 (±1.22) mm NHP: 0.75 (±0.88) mm	HP: MP: 27% pt; SP: 47.2% pt NHP: 10.7% pt	NR
Levin et al (2011) [32]	HP: MP: 15/447; SP: 55/1065 NHP: 23/747	NR	NR	NR	NR
Swierkot et al. (2012) [43]	HP: 6/149 NHP: 0/30	Mobility, fracture	NR	HP: 15/35 pt (43%), 39/149 imp. (26%) NHP: 2/18 pt (11%), 3/30 imp. (10%)	HP: 26/35 pt, 84/149 imp. (56%) NHP: 8/18 pt, 12/30 imp. (40%)
Roccuzzo et al. (2014) [40]	HP: MP: 3/96; SP: 3/102 NHP: 0/54	NR	*Imp*. *with BL* ≥ *3 mm* HP: MP: 9.4%; SP: 10.8% NHP: 0%	HP: MP: 52.2% pt; SP: 66.7% pt NHP: 18.8% pt	NR
Degidi et al. (2016) [29]	HP: 3/81 NHP: 5/203	NR	HP: 1 y: 0.89 mm (±0.17); 2 y: 1.07 mm (±0.18); 5 y; 1.48 mm (±0.27); 10 y: 2.01 mm (±0.27) NHP: 1 y: 0.83 mm (±0.21); 2 y: 1.02 mm (±0.22); 5 y: 1.40 mm (±0.29); 10 y: 2.79 mm (±0.34)	Overall: 16 (8.29%) imp. 14 (17.5%) pt HP: 13/55 imp. NHP: 3/139 imp.	Overall: 35 (18.13%) imp.; 26 (32.5%) pt
Roccuzzo et al. (2017) [42]	HP: 3/35 NHP: 1/33	NR	HP: 0.78 (±0.59) mm NHP: 0.43 (±0.5) mm	HP: 12/30 imp. (40%) NHP: 7/38 imp. (18.4%)	NR
Akram et al. (2019) [27]	HP: 4/48 NHP: 0/11	Mobility	HP: 0.76 mm in the first y after loading and 0.35 mm in the following third y NHP: 0.42 mm in the first y after loading and 0.14 mm in the following third y	NR	NR
Roccuzzo et al. (2022) [38]	HP: MP: 5/59; SP: 5/71 NHP: 2/39	NR	*Implants with BL* ≥ *3 mm* HP: MP: 33.3%; SP: 35.2% NHP: 17.9%	(*10–20 y F‐U*): HP: MP: 14 pt (48.3%); SP: 19 pt (61.3%) NHP: 7 pt (33.3%)	NR
Roccuzzo et al. (2023) [40]	NR	NR	NR	*Pt level*: 10 y: HP: 9^a^; 5^b^. NHP: 0^a^; 0^b^ 20 y: HP: 25^a^; 13^b^. NHP: 3^a^; 2^b^ *Imp*. *level*: 10 y: HP: 19^a^; 10^b^. NHP: 0^a^; 0^b^ 20 y: HP: 46^a^; 18^b^. NHP: 7^a^; 4^b^	*Pt level*: 10 y: HP: 40. NHP: 17 20 y: HP: 25. NHP: 15 *Imp*. *level*: 10 y: HP: 81. NHP: 29 20 y: HP: 54. NHP: 22

Abbreviations: AP, aggressive periodontitis; BL, bone loss; CP, chronic periodontitis; HP, history of periodontitis; Imp., implant; MP, moderate periodontitis; NHP, no history of periodontitis; NR, not reported; SP, severe periodontitis; y, year.

^a^ Defined as radiographic bone loss ≥ 3 mm.

^b^ Defined as radiographic bone loss ≥ 3 mm and probing depth ≥ 6 mm.

**TABLE 3:** Details regarding periodontal diagnostic criteria, case definitions for peri‐implantitis and peri‐implant mucositis, marginal bone loss (MBL) definition, and assessment methods.

Study	Periodontal diagnostic criteria	Case definition for peri‐implant mucositis	Case definition for peri‐implantitis	Definitions of MBL	Assessment methods for MBL
Karoussis et al. (2003) [31]	AAP 1999	NR	CIST protocol (Lang et al. 2000) for diagnosis and treatment	Distance from mesial and distal imp. shoulder and first clear BIC	Rx with customized Rinn film holder and rigid film–object–X‐ray source coupling to a beam aiming device. Rx captured with black and white camera and viewed on a light box, transferred to a PC, and digitized with a frame grabber hardware card. Digitized images stored with a resolution of (512 × 512 × 8) bit pixels (256 shades of gray), displayed on a monitor, and linear measurements performed with a cursor. Bone height changes calculated over the entire observation period and yearly
Mengel and Flores (2005) [34]	AAP 1999	NR	NR	Distance from marginal bone level to the top edge of the imp. expressed in relation to torsion section of the imp. (0.6 mm according to manufacturer)	Rx by one person as single‐tooth films with paralleling technique at baseline, immediately after insertion of the superstructure, and at 1 and 3 y. Each Rx was framed as a slide, digitized with 675 dpi resolution, and stored as a bitmap file. Digitized Rx evaluated by a software program by a blinded person
Mengel and Flores (2005) [35]	AAP 1999	NR	NR	Distance from alveolar crest to mesial and distal imp. shoulder expressed in relation to the torsion section of the imp. (0.6 mm according to manufacturer)	Rx by one person as single‐tooth films with paralleling technique at baseline, immediately after insertion of the superstructure, and at 1 and 3 y. Each intraoral Rx framed as a slide, digitized with 675 dpi resolution, and stored as a bitmap file. Digitized Rx evaluated by Digora software. BL determined metrically as a linear function, using the “distance measuring” function of the software. Rx evaluated by a person not involved in clinical investigation
Mengel et al. (2007) [36]	AAP 1999	NR	NR	Distance from the marginal bone level to the occlusal edge of the imp. expressed in relation to torsion section of the imp. (0.6 mm according to manufacturer)	Rx by one person as single‐tooth films with paralleling technique, immediately after insertion of the superstructure, and at 1 and 3 y, framed as a slide and digitized by a slide scanner with 675 dpi resolution. Digitized Rx evaluated by Digora software by a blinded person
Mengel et al. (2007) [33]	AAP 1989	NR	NR	Distance from the marginal bone to mesial and distal upper edge of the imp. related to imp. thread (0.6 mm according to manufacturer)	Rx by one person by parallel technique, immediately after insertion of the superstructure, and at 1, 3, 5, 8, and 10 y. Single‐film images framed as slides, digitized with a 675‐pixel slide scanner, stored as bitmap files and evaluated by a software
Gatti et al. (2008) [30]	PSR	NR	Loss of > 2 mm of peri‐implant marginal bone from last Rx assessment, with pus or another sign of infection and PD > 5 mm	Distance from coronal margin of imp. collar and the most coronal point of BIC	Marginal bone level changes on periapical intraoral Rx made with the paralleling technique between imp. loading and 5 y. Measurements made by a single nonblinded calibrated investigator with magnifying ocular grid of mesial and distal bone level to the nearest 0.1 mm
De Boever et al. (2009) [28]	AAP 1999	NR	NR	Distance from mesial and distal imp.–abutment junction and bone related to distance between two threads	MBL analyzed by one examiner on periapical Rx taken yearly by long cone technique, scanned to digital images and produced on a PC screen. Baseline and end point Rx were compared
Roccuzzo et al. (2010, 2012) [39, 41]	“S score”		NR	Distance between imp. shoulder and most coronal visible BIC measured in mm both at mesial and distal aspect of each imp	After crown/bridge cementation, baseline intraoral Rx obtained by the parallel long‐cone technique and film holder. Rx digitalized and measurements taken by a software. 10‐year values were compared with baseline values
Levin et al. (2011) [32]	AAP 1999	NR	NR	NR	NR
Swierkot et al. (2012) [43]	AAP 1999	PD ≥ 5 mm with BOP and no BL	PD > 5 mm with/without BOP and annual BL > 0.2 mm	Distance from marginal bone level to mesial and distal upper edge of imp. related to imp. thread	Standardized Rx taken by two persons by parallel technique, immediately after insertion of the superstructure and at 1, 3, 5, 10, and 15 y. Digitalized Rx evaluated with a software by an independent masked examiner
Roccuzzo et al. (2014) [37]	“S score”	CIST protocol (Mombelli & Lang 1998) for diagnosis and treatment		NR	NR
Degidi et al. (2016) [29]	NR	Reversible inflammatory reactions in the soft tissues, manifested as inflammation of mucosal cuff with edema, redness and BOP (Albrektsson & Isidor 1994)	Inflammatory reaction with supporting BL, manifested as infection with milky exudate and BL around the imp, as Rx translucency (Albrektsson & Isidor 1994)	Distance between mesial and distal imp.–abutment junction and the highest coronal bone point	Measurement rounded off to nearest 0.1 mm. A Peak Scale Loupe with a magnifying factor of 79 and a scale graduated in 0.1 mm were used. Measurements were taken mesially and distally and then averaged for each imp
Roccuzzo et al. (2017) [42]	NR	CIST protocol (Mombelli & Lang 1998) for diagnosis and treatment		Distance between mesial and distal imp. shoulder base and most coronal visible BIC No calibration method described	Standardized periapical intraoral films with a long cone technique
Akram et al. (2019) [27]	AAP 1999	NR	NR	Distance between mesial and distal machined bevel and edge of first microthread of imp. to most coronal bone	Digital periapical Rx with paralleling technique and film holders, immediately after insertion of the superstructure and at 1, 2, and 3 y, incorporated in a software (at 1:1 ratio) and examined on a calibrated computer display screen with an image analyzer
Roccuzzo et al. (2022) [38]	“S score”	CIST protocol (Lang et al. 1997; Mombelli & Lang 1998) for diagnosis and treatment		NR	NR
Roccuzzo et al. (2023) [40]	“S score”	≥ 1 BOP sites and/or suppuration with/without increased PD without BL beyond crestal changes from initial remodeling	BOP and/or suppuration, increased PD with BL beyond crestal changes from initial remodeling	Distance between mesial and distal imp. shoulder and most coronal visible BIC. Distance between two threads for calibration	Rx calibrated and evaluated using a software. To accurately identify true radiographic linear BL, the height of the supracrestal machined neck was subtracted from measured values. Measurements taken in duplicate by two experienced and calibrated examiners not involved in treatment or follow‐up examination

Abbreviations: AAP, American Academy of Periodontology; BIC, bone‐to‐implant contact; BOP, bleeding on probing; CIST, cumulative interceptive supportive therapy; Imp., implant; MBL, marginal bone loss; NR, not reported; PD, probing depth; PSR, periodontal screening and recording.

Different case definitions of peri‐implant diseases were adopted, such as the criteria reported for Cumulative Interceptive Supportive Therapy (CIST) (Lang et al. [47], Mombelli and Lang [48], Lang, Wilson and Corbet [49], or those provided by Albrektsson and Isidor [50]). Swierkot et al. [43] diagnosed peri‐implantitis in the presence of PD > 5 mm, with or without BOP, and annual bone loss > 0.2 mm. Gatti et al. [30] defined peri‐implantitis as loss of > 2 mm of peri‐implant marginal bone from the last radiographic assessment in the presence of pus or another sign of infection and PD > 5 mm. The current case definitions for peri‐implant mucositis and peri‐implantitis from the Consensus Report of the 2017 World Workshop on the Classification of Periodontal and Peri‐Implant Diseases and Conditions [44] were adopted only by Roccuzzo et al. [40] (see Table 3 for definitions).

### 3.6 Risk of Bias Assessment

Details regarding the risk of bias evaluation are reported in Table S4. Eight of the 14 included studies (11 articles) were considered at low risk of bias [28, 29, 30, 32, 37, 38, 39, 40, 41, 42, 43] as they obtained four stars in the Selection domain, one or two stars in the Comparability domain (only one study received 0 stars [42]) and two or three stars in the Results domain. The remaining studies resulted in a moderate risk of bias [27, 31, 33, 34, 35, 36] because they did not earn any stars in the Comparability domain. No study was at high risk of bias.

### 3.7 Quality of Evidence

The quality of evidence for primary and secondary outcomes ranged from nonsignificant to suggestive. In particular, the implant survival rate and MBL (overall) was found to have a suggestive quality of evidence. All the other outcomes showed weak to nonsignificant quality of evidence (Table S5).

### 3.8 Synthesis of the Results

#### 3.8.1 Implant Loss

**FIGURE 1:**
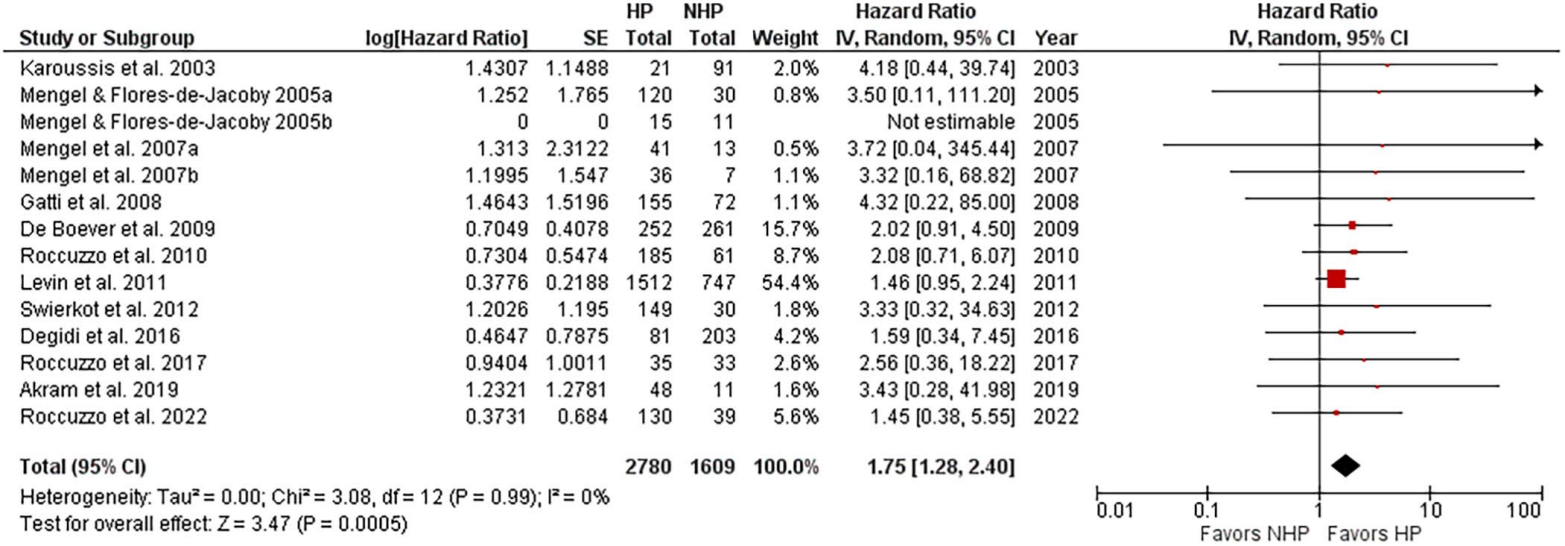
Overall risk of implant loss in HP versus NHP patients.

The forest plot of the implant loss (HR) comparing implants placed in NHP and HP patients is shown in Figure 1. An HR > 1 indicated a greater risk of implant loss for implants placed in the HP than in the NHP group. Data were analyzed at the implant level.

**FIGURE 2:**
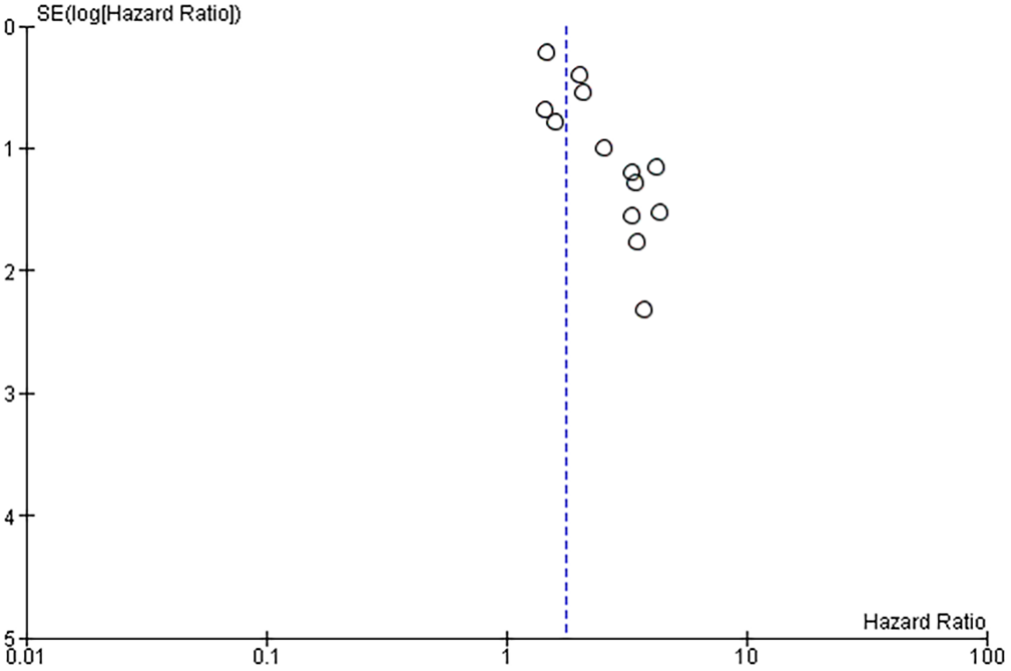
Visual representation of publication bias for the studies reporting the primary outcome event “implant loss.”

HP patients showed a significantly greater risk of implant loss, with an HR value of 1.75 (95% CI: 1.28–2.40; *p* = 0.0005) and a suggestive grade of evidence quality. The interstudy consistency was high (*I* ^2^ = 0%) and the funnel plot showed a certain symmetry in the distribution of studies reporting “implant loss” in the comparison between HP and NHP groups, indicating a low risk of publication bias for this outcome (Figure 2), as also quantified by Egger's test (*p* = 0.15; two‐tailed).

**FIGURE 3:**
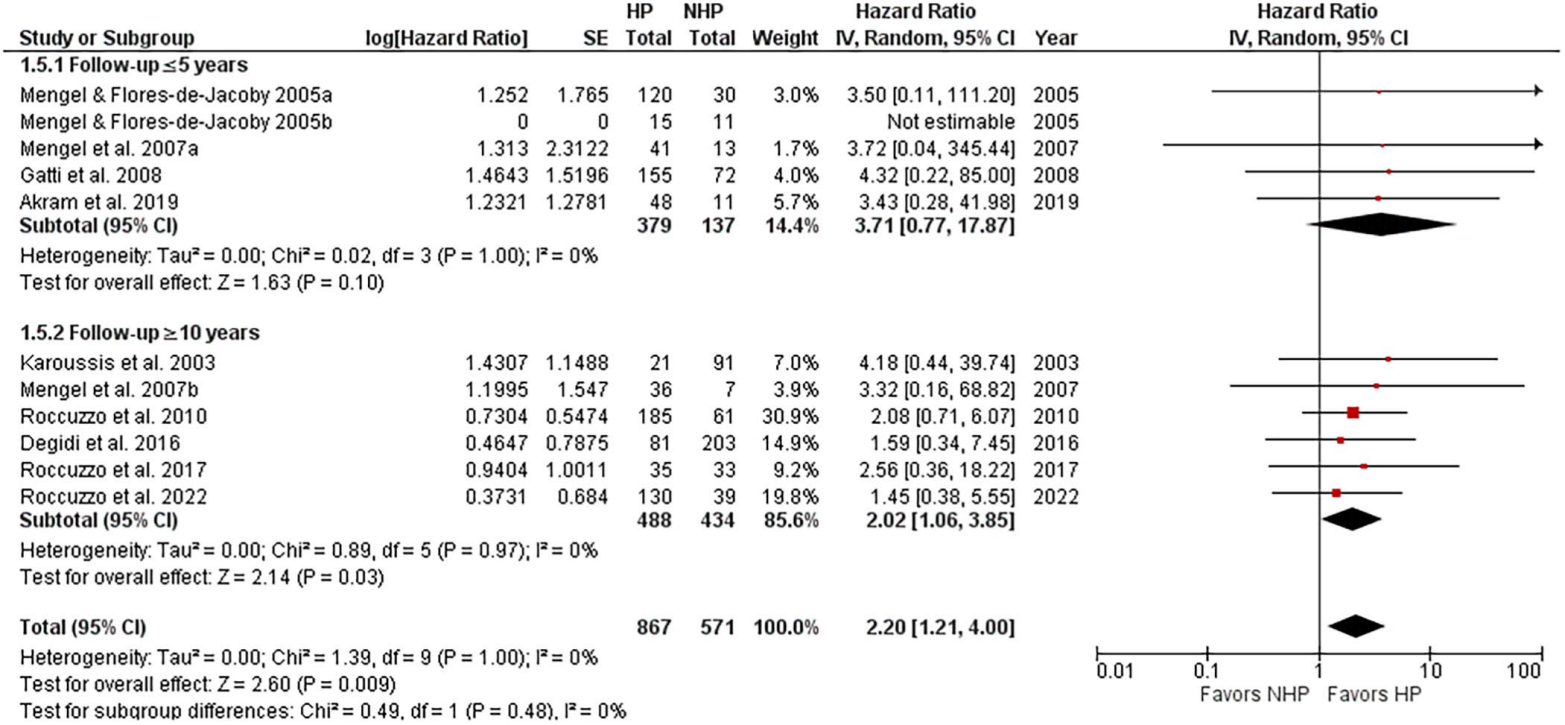
Subgroup analysis concerning the risk of implant loss in HP versus NHP patients for different follow‐up durations.

Studies with a definite follow‐up time duration were divided into two subgroups: follow‐up ≤ 5 years and ≥ 10 years. Interestingly, only the subgroup with a follow‐up duration of ≥ 10 years showed a greater risk for implant loss in HP compared to NHP patients (*p* = 0.03; 95% CI: 1.06–3.85) (Figure 3).

**FIGURE 4:**
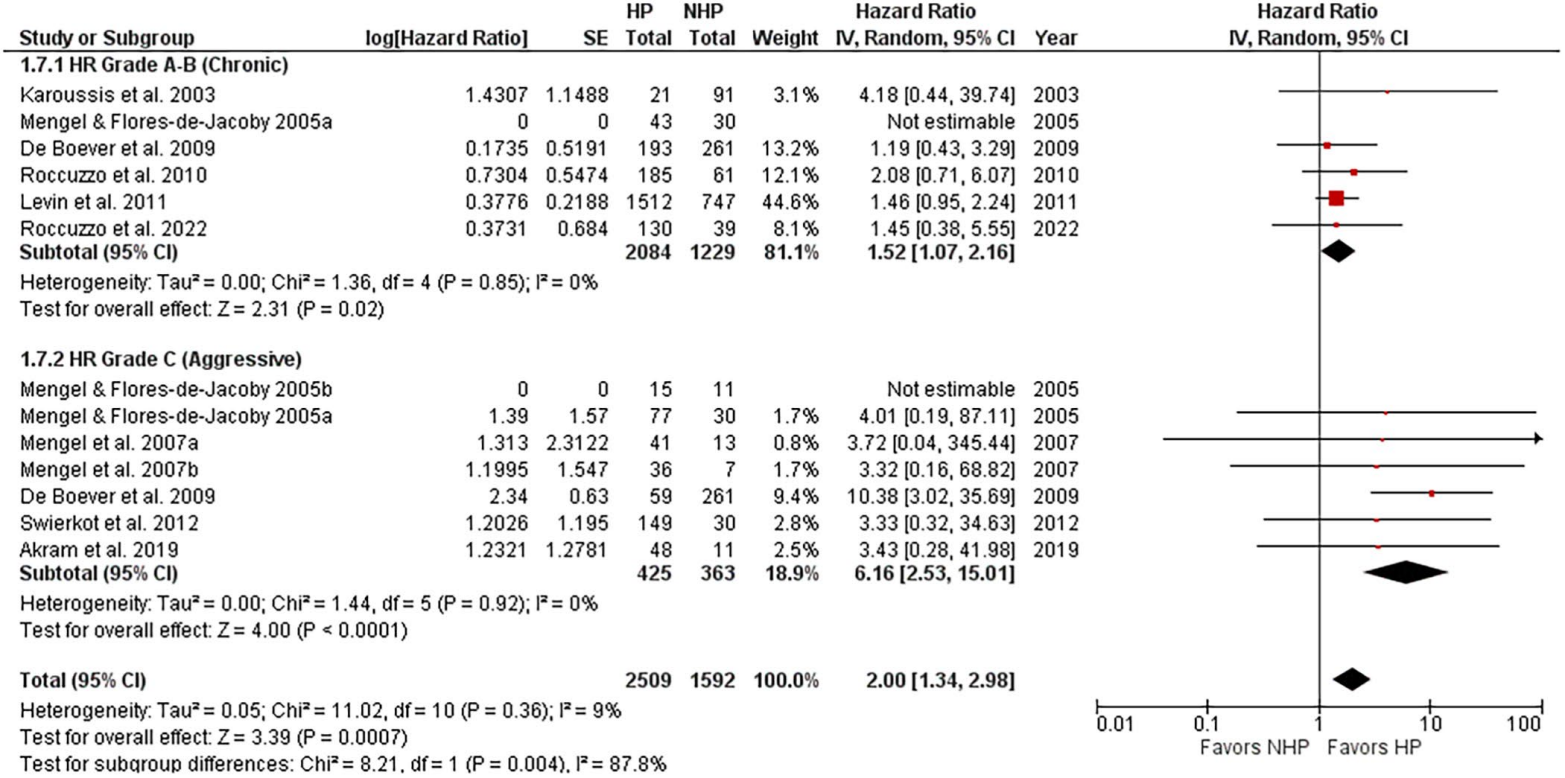
Subgroup analysis concerning implant loss risk in patients with a history of grades A–B (chronic) or grade C (aggressive) periodontitis compared to NHP patients.

A subgroup analysis was performed to investigate the effect of the rate of progression (chronic/aggressive or grades A–B/C following the current classification) and severity (moderate/severe or stages I–II/III–IV following the current classification) of periodontitis. Both subgroup studies on patients with a history of grades A–B (chronic) and grade C (aggressive) periodontitis showed a significantly greater risk of implant loss compared to the NHP group (Figure 4), significant (*p* = 0.004) subgroup differences, and a high intergroup heterogeneity (*I* ^2^ = 87.7%). Regarding the periodontitis stage, a subgroup analysis, due to the heterogeneity in the case definitions among the studies, was limited to only one study [32] in which patients with a history of stages III–IV (severe) periodontitis showed a statistically significant greater risk of implant loss compared to NHP patients (HR: 1.62, 95% CI: 1.03–2.54; *p* = 0.04), whereas patients with stages I–II (moderate) periodontitis showed a nonsignificantly greater risk of implant loss compared to NHP patients, with nonsignificant subgroup differences (Figure S2).

**FIGURE 5:**
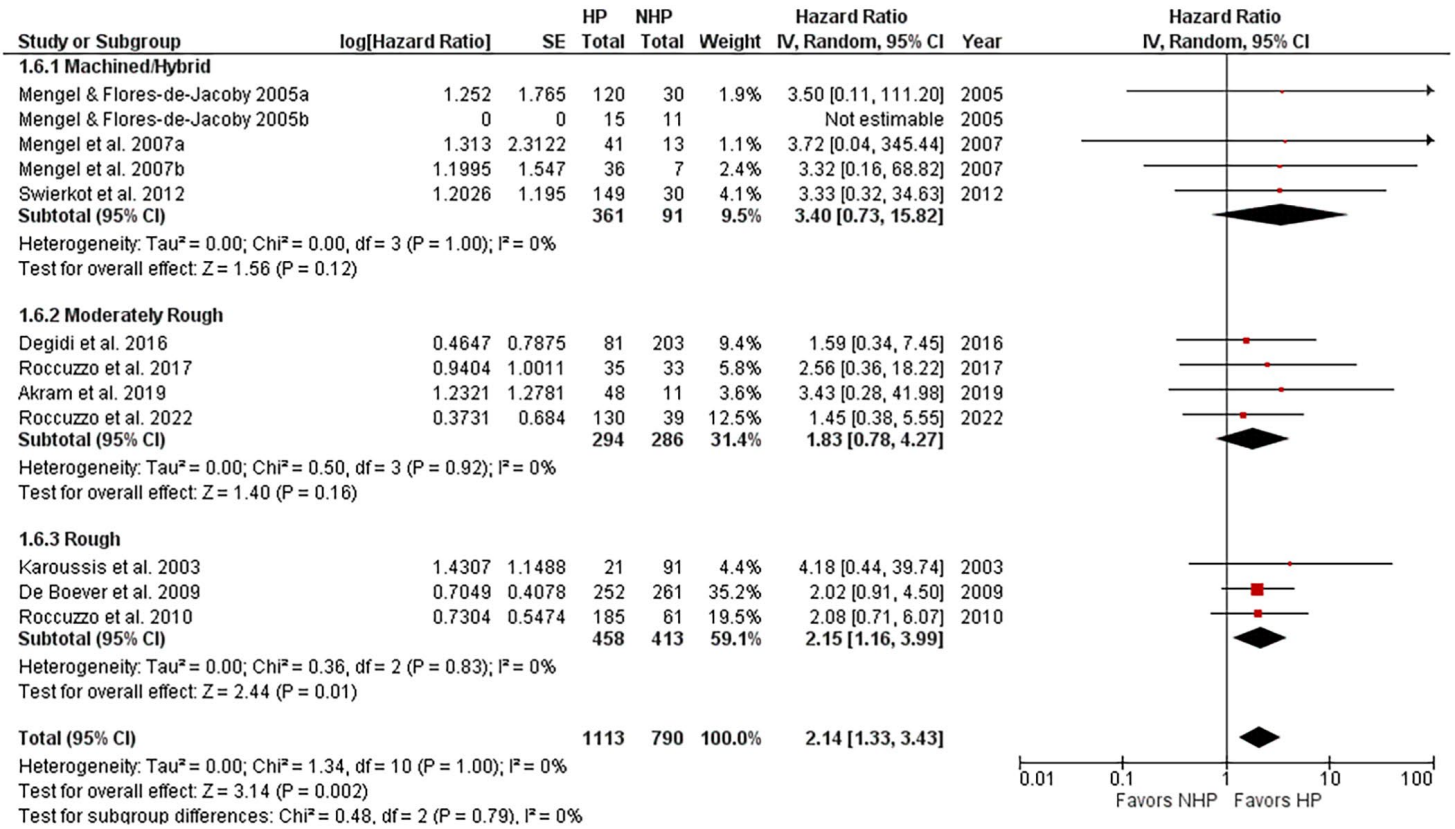
Subgroup analysis concerning the risk of implant loss in HP patients receiving implants with machined/hybrid, moderately rough, and rough surfaces compared to NHP patients.

A subgroup analysis concerning the type of implant surface was also performed. Studies that used machined and hybrid implant surfaces were grouped together, while modified implant surfaces were divided into two groups (moderately rough and rough) following the classification of Albrektsson and Wennerberg [51], only studies that used rough surfaces showed a significantly greater risk of implant loss in HP compared to NHP patients, with an HR of 2.15 (95% CI: 1.16–3.99; *p* = 0.01), while such a difference did not emerge for machined/hybrid and moderately rough surfaces (Figure 5). The test for subgroup differences revealed a nonsignificant effect (*p* = 0.79) with a low intergroup heterogeneity (*I* ^2^ = 0%).

Due to the lack of detail in the data reported by the included studies, it was not possible to conduct further subgroup analyses for other potential confounders or set up metaregressions for any of them. Sensitivity analyses were performed to investigate the homogeneity of the patient cohorts in terms of comorbidities, treatments, and baseline conditions. In particular, studies including patients with short implants [27], performing postextractive, immediate loading, or regenerative techniques [28, 29, 30, 35, 43], with overdenture rehabilitations [28, 30, 33, 36, 43], or with comorbidities [28, 32] were selectively excluded from the analysis without significant effect in the overall registered effect size (data not shown).

#### 3.8.2 Peri‐Implant Marginal Bone Loss

**FIGURE 6:**
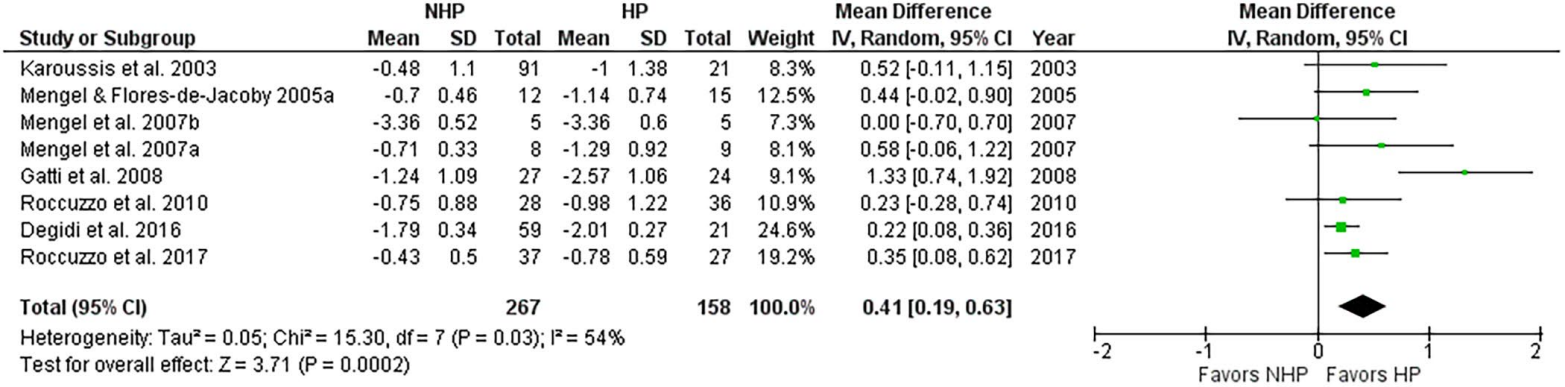
Mean difference in peri‐implant marginal bone loss between HP and NHP groups.

When mesial and distal MBL values were reported separately [31], mesial values were taken. When MBL data regarding grade C (aggressive) and grades A–B (chronic) periodontitis were separately provided, the former was considered [34]. All the studies considered prosthetic loading as the baseline for MBL measurements. The quantitative analysis of pooled data revealed in the HP group significantly greater MBL, with a DM of 0.41 mm (95% CI 0.19, 0.63; *p* = 0.0002) with a suggestive grade of evidence quality, and a moderate interstudy heterogeneity (*I* ^2^ = 54%) (Figure 6). Subgroup analysis for follow‐up, rate of progression, and implant surface characteristics did not reveal significant subgroup differences (Figures S3A–C).

#### 3.8.3 Peri‐Implant Mucositis

**FIGURE 7:**
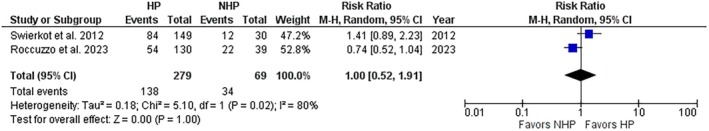
Risk of peri‐implant mucositis at implant level in HP versus NHP patients.

HP and NHP groups showed a comparable risk of developing peri‐implant mucositis, measured both at implant level (Figure 7) and patient level (Figure S4), with high heterogeneity. Due to the paucity of available studies, no subgroup analysis was performed.

#### 3.8.4 Peri‐Implantitis

**FIGURE 8:**
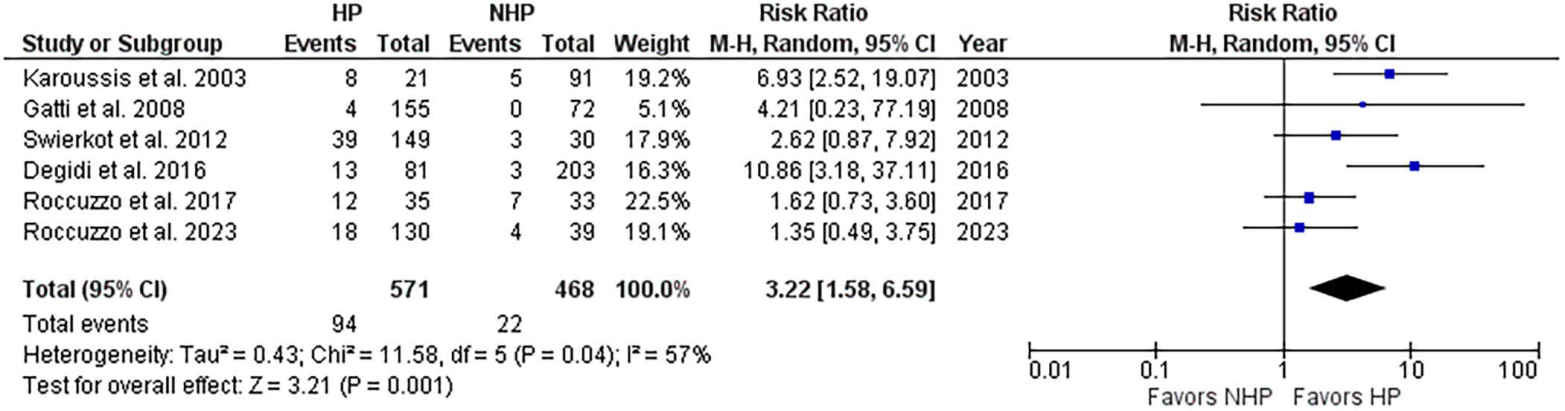
Risk of peri‐implantitis at implant level in HP versus NHP patients.

Peri‐implantitis measured at implant level showed a significantly greater rate in HP compared to NHP patients, with a RR of 3.24 (95% CI: 1.58–6.64; *p* = 0.001; *I* ^2^ = 57%) (Figure 8), with a nearly suggestive grade of evidence quality, and a moderate interstudy heterogeneity. Similar results emerged from the analysis at the patient level (Figure S5). Subgroup analysis for follow‐up, rate of progression, and implant surface characteristics did not reveal significant subgroup differences at both patient and implant levels. A trend toward a significantly greater risk for the HP group for the ≥ 10‐year follow‐up (compared to ≤ 5‐year follow‐up) and for rough surfaces (compared to machined/hybrid and moderately rough) subgroups was found (Figures S6 and S7), though the strength of the evidence is impaired by the reduced number of studies included in these analyses.

## 4 Discussion

The results of the present meta‐analysis support, with suggestive evidence, the association between a HP and a greater risk of implant loss over time.

The overall risk of implant loss in HP patients appeared to be 1.75 times greater than in the NHP group, with high interstudy consistency, low risk of publication bias, highly significant overall effect, and suggestive quality of evidence. These results confirmed what was shown by other meta‐analyses, where similar findings have been reported [14, 19, 20, 52]. The number of studies (14) included in our meta‐analysis for this outcome, as well as their nature of prospective studies, further provide strength to this evidence. Among the other available high‐quality systematic reviews of prospective studies published on the same topic, Stacchi et al. [15] included fewer (only three) studies, and failed to find a statistically significant difference between HP and NHP patients for implant loss. Serroni et al. [20] reached a similar result (with a risk of implant loss 1.74 greater for the HP group than the NHP one)[41], but with a lower number of studies (12), despite the inclusion also of shorter (≤ 3 years) follow‐up studies.

The subgroup analysis performed on implant loss according to different follow‐up periods confirmed the importance of such a variable on the risk of implant loss over time, in line with the recent meta‐analyses of Carra et al. [19], and Serroni et al. [20], but also with some differences due to the inclusion criteria applied and to a different grouping of the studies analyzed. In particular, Carra et al. [19], who also included nonprospective studies, showed a significantly different risk between HP and NHP patients for the ≥ 5‐year and 10‐year follow‐up subgroups, but not a significant difference for the < 5‐year follow‐up studies, with a nonsignificant test for subgroup differences. Serroni et al. [20] performed three separate analyses for ≤ 5‐year follow‐up studies, > 5‐year follow‐up studies (in which also 10‐ and 20‐year studies were included), and 10‐year follow‐up studies (in which 10‐year studies were grouped separately). No subgroup analysis was performed, thus no test for subgroup differences was applicable. They found a significantly greater risk for HP versus NHP patients for all three groups of studies and a larger effect size for > 5‐year and 10‐year follow‐up studies. In this study, the subgroup analysis performed, in which studies with a not well‐defined follow‐up were excluded, a significant difference for the ≥ 10‐year follow‐up subgroup, but not for ≤ 5‐year follow‐up subgroup, was found, though with a nonsignificant test for subgroup differences. Discrepancies with previous meta‐analyses are mainly due to the exclusion of studies with a mean, instead of defined, follow‐ups. This finding further underlines the importance of a longer follow‐up to detect a significant difference in implant loss between HP and NHP groups, thus possibly moving the threshold beyond the 5‐year limit proposed by Carra et al. [19].

Regarding the severity of periodontitis experienced by patients, only those with a history of stages III–IV (severe) periodontitis showed a significantly greater risk of implant loss compared to NHP patients, whereas no significant difference was found comparing patients with a history of stages I–II (moderate) periodontitis and NHP. It must be noted, however, that this finding is based on a single, though large, study [32], due to the exclusion of other studies adopting different case definitions, and this aspect inevitably affects the strength of the evidence [54]. Only the study of Levin et al., indeed, followed the AAP 1999 classification [32], whereas Gatti et al. applied the PSR score [30] and Roccuzzo et al. the “S score” [37, 38, 39, 40, 41], both of them referring to pocket depth instead of clinical attachment level.

This study showed a particularly greater risk of implant loss (HR 6.16) in patients with a history of grade C (aggressive) periodontitis versus NHP patients than in patients with a history of grades A–B (chronic) periodontitis versus NHP patients (HR 1.53). None of the studies considered was recent enough to refer to the current 2018 AAP/EFP classification [55]; however, we have chosen to update the terminology used to describe our results. All the included primary studies referred to the AAP 1999 classification of periodontal diseases [56], including the studies of Roccuzzo et al. [37, 38, 39, 40, 41] in which the authors, though not mentioning that classification, exclude patients with aggressive periodontitis and the study of Mengel, Behle, Flores‐de‐Jacoby 2007 [33], that follows the AAP 1989 classification referring to “rapidly progressing periodontitis.” Sgolastra et al. [14] performed similar subgroup analyses in their systematic review, though basing their finding on a lower number of studies selected by different inclusion/exclusion criteria, reporting similar results. Based on this finding, we could speculate that the greater the grade (rate of progression) of the periodontitis experienced by implant patients, the greater the risk of implant loss over time, as also confirmed by a recent 10‐year retrospective study [57] reporting a greater rate of implant failure due to peri‐implantitis in grade C versus A–B periodontitis patients.

A recent systematic review concluded that a history of periodontitis, even under regular supportive postimplant treatment, remains a negative indicator for implant survival in rough‐surfaced but not in smooth‐surfaced implants [58]. In this meta‐analysis, in line with the abovementioned study, the risk of implant loss was shown to be significantly greater in HP versus NHP patients with rough implants, whereas such a risk was not different in HP versus NHP patients rehabilitated by smooth/hybrid implants or moderately rough implants (and a similar trend was found also for the peri‐implantitis outcome). These findings, however, needs to be cautiously interpreted. No significant effect, indeed, of the test for subgroup differences was found. The limited number of studies and the heterogeneity of the subgroups, for example, in terms of follow‐up time and specific surface treatment adopted, may limit the strength of evidence. Furthermore, some of these studies, for example, that by Karoussis et al. [31], De Boever et al. [28], and Roccuzzo et al. [41] employing TPS implants, have mainly a historical value nowadays, thus reducing the generalizability of the findings. Nevertheless, they have the merit to support the concept, though to be confirmed by further studies that the use of very rough surfaces (some of which are still on the market today) in HP patients may expose them to a higher risk of failure. They give strength to a topic, the effect of implant surface characteristics on implant prognosis, that is still a matter of debate.

The present analysis showed that the risk of peri‐implantitis occurrence is about 3.24 times greater in HP groups compared to NHP (at both implant and patient level), in line with other data reported in the literature [14, 19, 20, 52] with a risk of 2.17–4.09 times greater in HP versus NHP patients. Conversely, our meta‐analysis did not find differences between HP and NHP patients in terms of peri‐implant mucositis rate, though such a finding is supported by only two studies adopting different case definitions of the disease and needs to be cautiously considered.

Also, a significantly greater MBL in HP compared to NHP patients was demonstrated in the present analysis (difference in mean—DM of 0.41 mm), in line with what was reported by other similar meta‐analyses, where DM values ranging from 0.44 to 0.75 mm were found [14, 20, 52, 58].

Despite the highly significant differences found, the strength of the evidence for both these secondary outcomes is inevitably reduced compared to the implant loss outcome. This is mainly due to the lower number of studies and the great variability in methods for MBL measurements, with the frequent absence of a calibration procedure, and in case definitions of peri‐implant diseases. Furthermore, both patient and implant level data were pooled together for MBL meta‐analysis, and, for both MBL and peri‐implantitis, results at different follow‐ups were analyzed together.

It must be underlined that several potential confounders may impact implant outcomes and such an effect, despite being downsized by pooled data analyses and sensitivity analyses, cannot be totally excluded. The inclusion, indeed, of studies that are heterogeneous in terms of study setting (e.g., private practice or university) baseline characteristics of patients (e.g., smoking habit, compliance to SPC/SPIC, comorbidities), type of implants (e.g., brand, surface), surgical and prosthetic protocols (e.g., one/two stages, the timing of placement and loading, the implementation of bone regeneration), implant site location (e.g., upper or lower arch), and type of restorations (e.g., design, extension, material) contribute to a more realistic framework in favor of the effectiveness assessment of implant therapy, but it inevitably ends to affect the association between the exposure and the outcome.

The cardinal role of regular supportive peri‐implant care to maintain peri‐implant health and promote implant survival has been emphasized in the recent literature [10, 11, 53, 59]. The 2023 EFP S3 level clinical practice guideline for prevention and treatment of peri‐implant diseases [6] firmly recommends a guideline‐conformed treatment of periodontal diseases to a stable endpoint and adherence to a supportive periodontal care program (SPC) prior to implant placement as a form of primordial prevention of peri‐implant diseases. Furthermore, in patients rehabilitated with implants, a regular SPIC is strongly recommended for primary prevention of peri‐implant diseases [60]. The lack of compliance to the SPIC program has been suggested as one of the main factors worsening implant prognosis. It may act synergically with other risk factors, such as a history of periodontitis, but it also may act as a confounder. Patients with an HP, indeed, even if highly compliant to SPIC, may be associated with a high rate of peri‐implant diseases, as highlighted by a recent observational study reporting a high prevalence of peri‐implant diseases also in highly compliant patients with a history of advanced (stages III–IV) periodontitis [61]. All the studies included in this literature review report that implant therapy was delivered after complete periodontal treatment and that patients were all put in SPIC. Only three of them (five articles) [32, 37, 38, 39, 41], however, gave information about the compliance of patients to SPIC, and just two studies (three articles) [37, 38, 39, 41] correlate the compliance to SPIC with implant prognosis. In particular, Roccuzzo et al. in their studies reported a significantly greater proportion of patients with implant loss and with ≥ 3 mm bone loss in noncompliant than compliant patients, in the HP group, but not in the NHP one [39], treated with solid screw highly rough implants at 10 years of follow‐up. Although not statistically significant, the authors report the same trend (implant‐level), with moderately rough implants at 20 years in both HP and NHP groups in their 2022 study [37]. Methodological heterogeneity does not allow to meta‐analyze these two datasets. Further similar studies, however, possibly with higher numbers and rigorous selection of patients, will be essential for better comprehending the correlation between a history of periodontitis and implant prognosis net of possible confounders. This should be important to understand to what extent the occurrence of peri‐implant diseases and implant loss is due to worse compliance to SPIC or, actually, to a common ground of host susceptibility for periodontal and peri‐implant diseases, as well as to other variables (e.g., implant characteristics, residual periodontal lesions).

Although smoking habit has been widely investigated as a potential factor affecting dental implant outcomes, there is currently no conclusive evidence that constitutes a risk factor/indicator for peri‐implantitis, probably due to the frequent presence of background confounders (e.g., history of periodontitis itself) and to the differences in categorization of smokers and nonsmokers [10, 11]. De Boever et al. [28] found a reduced (though in a nonsignificant way) survival rate in patients with a history of aggressive periodontitis who were actual and former smokers compared to nonsmokers. In the study from Levin et al. [32], a lower implant SR was found in smokers compared to nonsmokers patients, especially at long‐term follow‐up (> 50 months). Also in the study from Degidi et al. [29], seven of eight failed implants were placed in smoker patients, thus, smoking was found to be statistically associated with implant failure. Furthermore, Swierkot et al. [43] showed that also the incidence of biological complications was greater in smoker versus nonsmokers patients.

### 4.1 Strengths

Several points of strength of this study can be pointed out in comparison with recently published systematic reviews on the same topic. Including prospective cohort studies, in a number higher than any other previously published systematic review on this topic, contributes to increasing the strength of the association between exposure and outcomes. The choice of hazard ratio, instead of risk ratio or odds ratio, to quantify the overall effect size for implant loss was chosen to include information on the temporal progression of the events within groups, since multiple follow‐up studies were included and information about the time of implant failure could be generally extracted or calculated. A quantitative assessment of factors potentially affecting implant outcomes in HP compared to NHP patients, such as follow‐up period, stage and grade of periodontitis, and implant surface characteristics, was carried out by subgroup analyses. Furthermore, a quantitative analysis of an outcome not assessed in previous similar studies, such as the peri‐implant mucositis occurrence in NHP and HP patients, could be carried out. Finally, the quality assessment of the included studies in terms of risk of bias and quality of evidence evaluation contributed to a more comprehensive interpretation of the findings and formulation of conclusions.

### 4.2 Limitations

The main weak point of the evidence emerging from this systematic review is due to the great methodological heterogeneity among the studies. The case has a great variety of definitions of periodontitis and peri‐implantitis. Furthermore, differences were found in the primary studies in terms of outcomes assessment and report; for example, not only MBL was measured in different ways, but also peri‐implantitis case definitions greatly varied among the included studies, thus limiting the strength of the emerging evidence. A great heterogeneity also in terms of potential confounding factors was found, as mentioned earlier, for the compliance to supportive periodontal treatment or the smoking habit, and only in a few cases were they duly reported so that the analysis of their possible influence on implant outcomes, beyond limited sensitivity analyses, could not be carried out. Furthermore, it must be considered that 6 of 14 of the included studies showed a moderate risk of bias, potentially affecting the reliability of the reported results. Although the publication bias was visually and statistically evaluated by Funnel plot and Egger's test, the possibility that studies with statistically significant results are more likely to be published, potentially skewing the overall conclusions [62], especially with small sample size studies, cannot be excluded. Additionally, it should be considered that only articles in English were included, and the majority of the studies were conducted in European countries, thus a geographic bias could limit the generalization of the findings.

## 5 Conclusions

Within the limits of heterogeneous case definitions and methods of assessment, a history of periodontitis significantly increases the risk for implant loss, particularly at long follow‐up (≥ 10 years) and in case of rapidly progressive forms (grade C), and for MBL and peri‐implantitis.

### 5.1 Implication for Clinical Practice

Patients and clinicians must be aware of the risk of the worst implant prognosis with a history of periodontitis, to suitably control risk factors and carry out the appropriate periodontal and implant treatment plan, implementing every effort to prevent any recurrence of periodontitis and any occurrence of peri‐implant complications and failures.

### 5.2 Implications for Research

Further well‐designed prospective studies with larger sample sizes and longer follow‐up, also taking account of the current case definition and classification of periodontal and peri‐implant diseases, are needed to strengthen the available scientific evidence, limiting study heterogeneity as well as the influence of possible confounding factors.

## Author Contributions

Marco Annunziata, Gennaro Cecoro, Massimo del Fabbro, and Luigi Guida conceived the concept/design. Marco Annunziata and Gennaro Cecoro conducted the data collection. Marco Annunziata, Gennaro Cecoro, Agostino Guida, Roberto Sorrentino, Massimo del Fabbro, and Luigi Guida were involved in the analysis/interpretation of the data. Marco Annunziata, Gennaro Cecoro, Gaetano Isola, and Paolo Pesce drafted the manuscript. All the authors critically revised the manuscript and approved the final version to be published.

## Conflicts of Interest

The authors declare no conflicts of interest.

## Protocol and Registration

This systematic review was previously registered in PROSPERO under registration number CRD42021264980.

## Supporting information


Data S1.

## Data Availability

The data that support the findings of this study are available from the corresponding author upon reasonable request.
