# Supplementary material for: Effectiveness of Implant Therapy in Patients With and Without a History of Periodontitis: A Systematic Review With Meta‐Analysis of Prospective Cohort Studies
Source: J Periodontal Res. 2024 Oct 28;60(6):524–43. doi: 10.1111/jre.13351 (PMC12312821; doi:10.1111/jre.13351)
Supplement: Supplementary file 1 — Data S1. [file JRE-60-524-s001.docx]

**Effectiveness of implant therapy in patients with and without a history of periodontitis: a systematic review with meta-analysis of prospective cohort studies**

**Supplementary Material**

Supplementary Tables

**Table S1.** Detailed search strategy for electronic databases.

| **Database** | **Search string** |
| --- | --- |
| Pubmed (MEDLINE) | ((dental implant[MeSH Terms]) OR (dental implantation[MeSH Terms])) AND (periodontitis OR (periodontal disease)) |
| Clinicaltrials.gov | *Condition or disease*: dental implants OR dental implantation  *Other terms*: periodontitis OR periodontal disease |
| Embase | ('dental implant' OR 'dental implantation') AND (periodontitis OR 'periodontal disease') |

**Table S2.** Criteria for quality of evidence classification in observational studies.

| **Category** | **Criteria** |
| --- | --- |
| Convincing, class I | - p < 1x10-6 - I2 < 50 % - 95 % prediction interval excluding the null - No small study effects and publication bias - Largest study with a statistically significant effect - No excess significance bias |
| Highly suggestive, class I | - p < 1x10-6   Largest study with a statistically significant effect |
| Suggestive, class III | - p < 1x10-3 |
| Weak, class IV | - p < .05 |
| Non-significant | - p > .05 |

**Table S3.** Excluded studies and reason for exclusion.

| **Study** | **Exclusion reason** |
| --- | --- |
| Grunder et al. 1999 (11) | Unclear definition of HPP group (reason 1) |
| Polizzi et al. 2000 (16) | Unclear definition of HPP group (reason 1) |
| Brägger et al. 2001 (4) | PHP/HPP number not available (reason 2) |
| Astrand et al. 2004 (2) | PHP/HPP number not available (reason 2) |
| Karoussis et al. 2004 (12) | PHP/HPP number not available (reason 2) |
| Fransson et al. 2005 (7) | PHP/HPP number not available (reason 2) |
| Màximo et al. 2008 (13) | PHP/HPP number not available (reason 2) |
| Wahlstrom et al. 2010 (20) | PHP/HPP number not available (reason 2) |
| Zetterqvist et al. 2010 (21) | PHP/HPP number not available (reason 2) |
| Corbella et al. 2011 (5) | PHP/HPP number not available (reason 2) |
| Akoglu et al. 2011(1) | PHP/HPP number not available (reason 2) |
| Rodrigo et al. 2012 (17) | PHP/HPP number not available (reason 2) |
| Degidi et al. 2012 (6) | PHP/HPP number not available (reason 2) |
| Pjetursson et al. 2012 (15) | PHP/HPP number not available (reason 2) |
| Schropp et al. 2013 (18) | PHP/HPP number not available (reason 2) |
| Frisch et al. 2015 (8) | PHP/HPP number not available (reason 2) |
| Gabay et al. 2015 (9) | PHP/HPP number not available (reason 2) |
| Uribarri et al. 2017 (19) | Unclear Study design (reason 3) |
| Göthberg et al. 2018 (10) | PHP/HPP number not available (reason 2) |
| Mohanty et al. 2018 (14) | Unclear definition of PHP group (reason 4) |
| Borisenko et al. 2020 (3) | Unclear definition of HPP group (reason 1) |

**Table S4.** Risk of bias in included studies (history of periodontitis): results of Analysis with the Newcastle-Ottawa Scale. ^a^ Five-year follow-up. A star system is used to design a semiquantitative assessment of study quality so that the highest quality studies are awarded a maximum of one star for each item except for those relating to comparability with the assignment of two stars. A maximum of nine stars can be allotted if all the above items are satisfied. A low risk of bias was considered for those studies scoring 7 to 9 stars, medium risk for those scoring 4 to 6 stars and high risk for those scoring <4 stars.

|  | **Selection** | **Comparability** | **Outcome** | **Total** |
| --- | --- | --- | --- | --- |
| Karoussis et al. 2003 | *** | 0 | ** | 5 |
| Mengel & Flores-de Jacoby 2005a | **** | 0 | ** | 6 |
| Mengel & Flores-de-Jacoby 2005b | **** | 0 | ** | 6 |
| Mengel et al. 2007a | **** | 0 | ** | 6 |
| Mengel et al. 2007b | ** | 0 | *** | 5 |
| Gatti et al. 2008 | **** | * | *** | 8 |
| De Boever et al. 2009 | **** | ** | ** | 8 |
| Roccuzzo et al. 2010, 2012 | **** | * | *** | 8 |
| Levin et al 2011 | **** | ** | ** | 8 |
| Swierkot et al. 2012 | **** | ** | *** | 9 |
| Roccuzzo et al. 2014, 2022, 2023 | **** | * | *** | 8 |
| Degidi et al. 2016 | **** | * | *** | 8 |
| Roccuzzo et al. 2017 | **** | 0 | *** | 7 |
| Akram et al. 2019 | ** | 0 | ** | 4 |

**Table S5.** Quality of evidence evaluation. IL: implant loss; MBL: marginal bone loss; PI: peri-implantitis

| **Outcome** | **Quality of evidence** |
| --- | --- |
| IL overall | Suggestive |
| MBL overall | Suggestive |
| Mucositis implant level | Non-significant |
| Mucositis patient level | Non-significant |
| PI implant level | Weak |
| PI patient level | Weak |

Supplementary Figures

**Figure S1.** PRISMA 2020 flow-chart of the selection process.


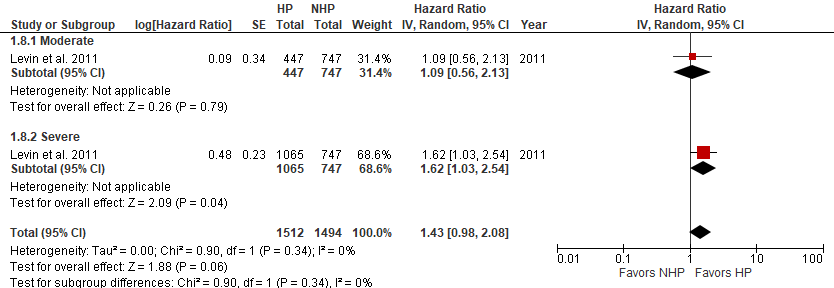


**Figure S2.** Subgroup analysis concerning the risk of implant loss in patients with a history of Moderate or Severe periodontitis compared to NHP patients.

a)
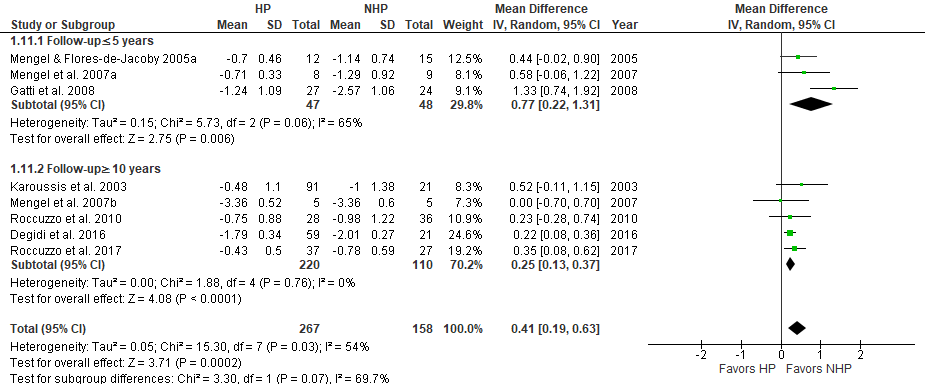


b)
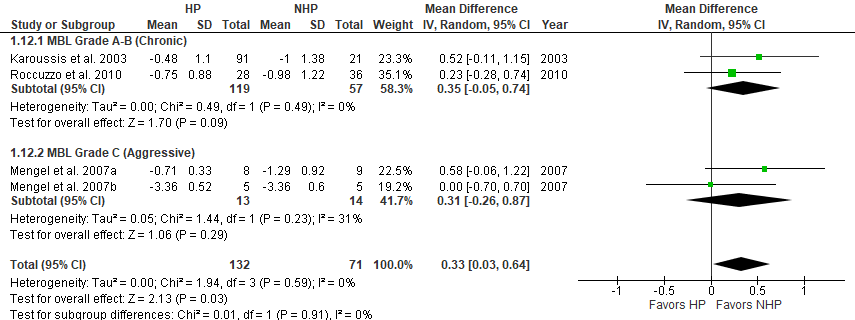


c)
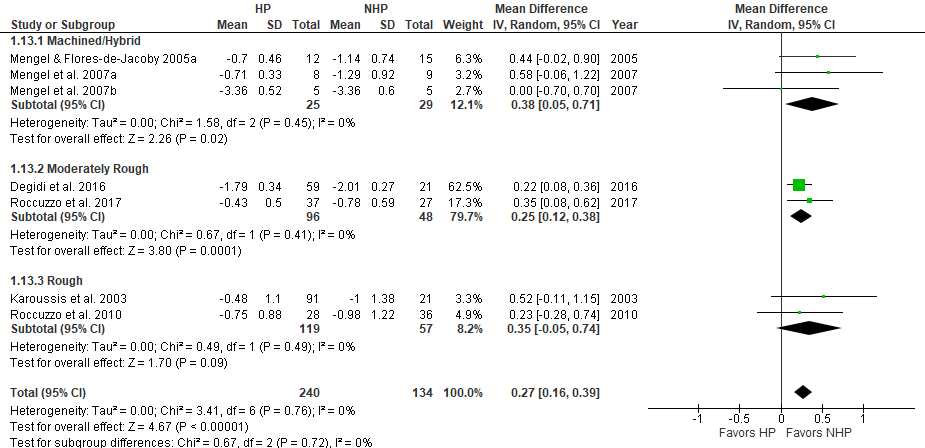


**Figure S3.** Subgroup analysis concerning peri-implant marginal bone loss for different follow-up durations (a), periodontitis rate of progression (b), and implant surface (c).


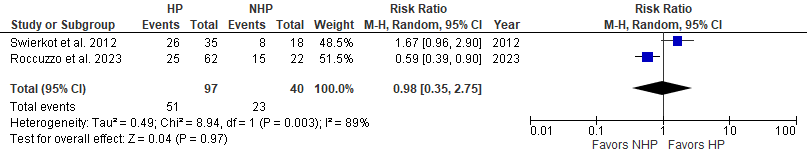


**Figure S4.** Peri-implant mucositis rate patient level.


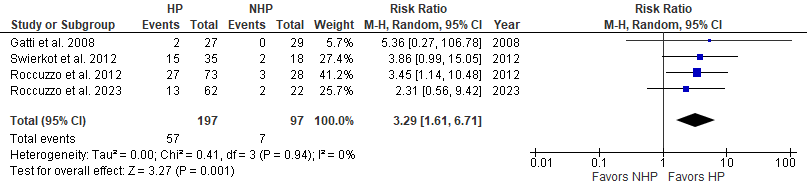


**Figure S5.** Peri-implantitis rate patient level.

a)
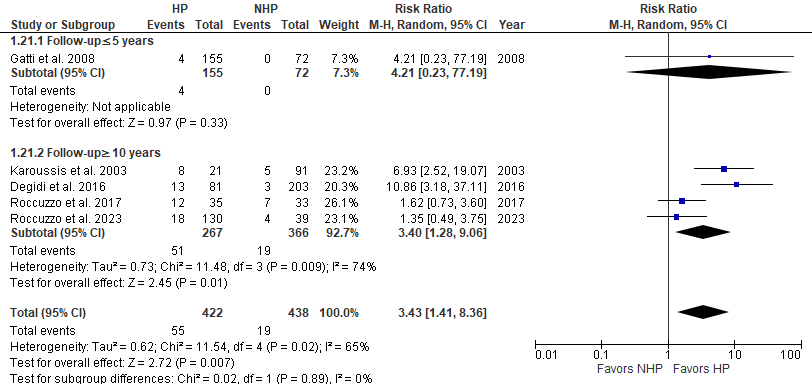


b)
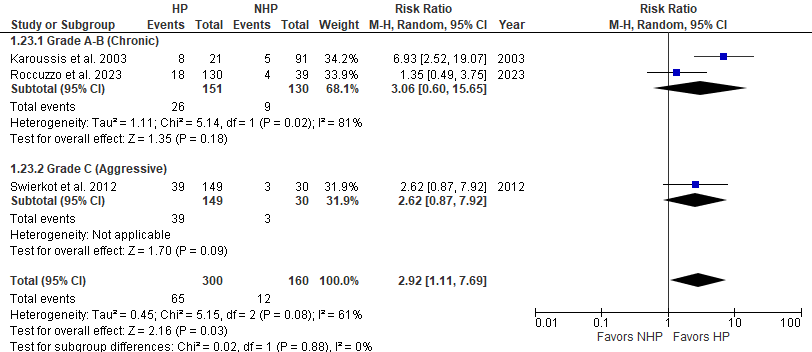


c)
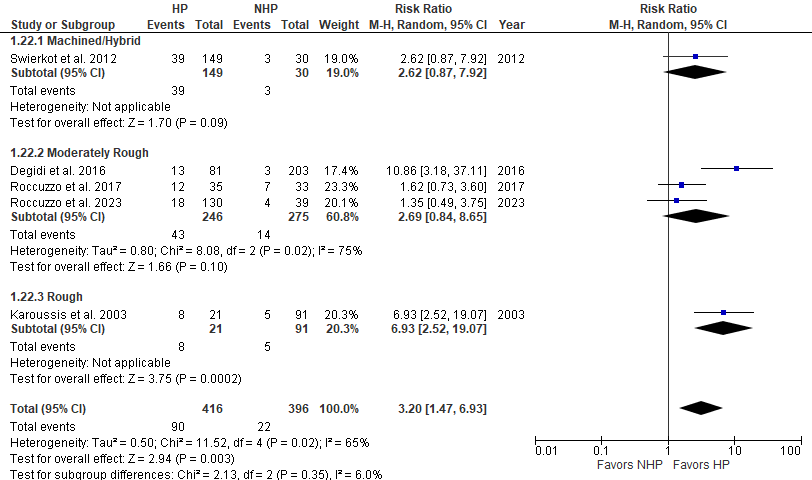


**Figure S6.** Subgroup analysis concerning peri-implantitis rate at implant level for different follow-up durations (a), periodontitis rate of progression (b) and implant surface (c).

1.
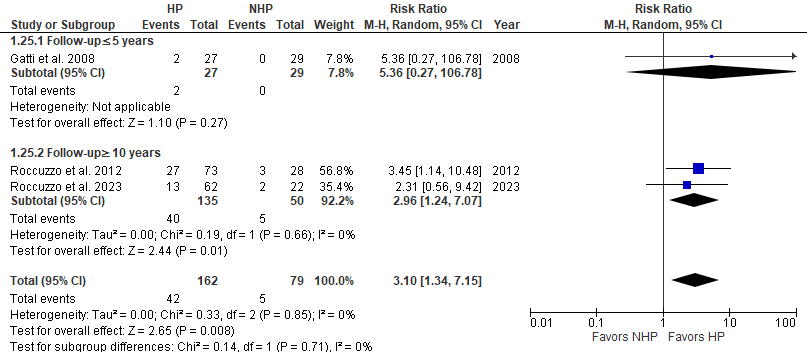

2.
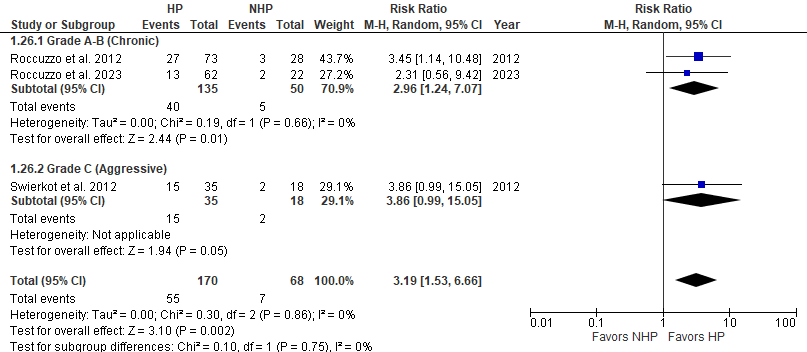

3.
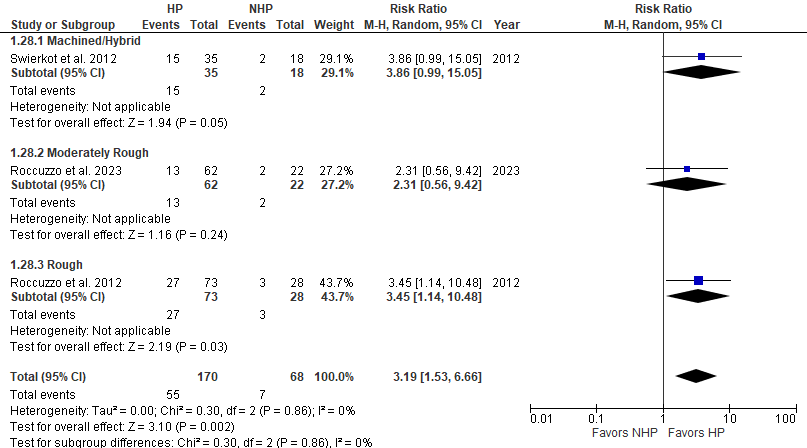


**Figure S7.** Subgroup analysis concerning peri-implantitis rate at patient level for different follow-up durations (a), periodontitis rate of progression (b) and implant surface (c).

**REFERENCES**

1. Akoglu B, Ucankale M, Ozkan Y, Kulak-Ozkan Y. Five-year treatment outcomes with three brands of implants supporting mandibular overdentures*Int J Oral Maxillofac Implants* 2011; 26: 188–194.

2. Astrand P, Engquist B, Anzén B, *et al.* A three-year follow-up report of a comparative study of ITI Dental Implants and Brånemark System implants in the treatment of the partially edentulous maxilla*Clin Implant Dent Relat Res* 2004; 6: 130–141.

3. Borisenko A, Antonenko M, Zelinsky N, Stolyar V, Popov R. EARLY POSTOPERATIVE COMPLICATIONS IN DENTAL IMPLANT PATIENTS*Georgian Med News* 2020; 23–28.

4. Brägger U, Aeschlimann S, Bürgin W, Hämmerle CH, Lang NP. Biological and technical complications and failures with fixed partial dentures (FPD) on implants and teeth after four to five years of function*Clin Oral Implants Res* 2001; 12: 26–34.

5. Corbella S, Del Fabbro M, Taschieri S, De Siena F, Francetti L. Clinical evaluation of an implant maintenance protocol for the prevention of peri-implant diseases in patients treated with immediately loaded full-arch rehabilitations*Int J Dent Hyg* 2011; 9: 216–222.

6. Degidi M, Nardi D, Piattelli A. 10-year follow-up of immediately loaded implants with TiUnite porous anodized surface*Clin Implant Dent Relat Res* 2012; 14: 828–838.

7. Fransson C, Lekholm U, Jemt T, Berglundh T. Prevalence of subjects with progressive bone loss at implants*Clin Oral Implants Res* 2005; 16: 440–446.

8. Frisch E, Ziebolz D, Vach K, Ratka-Krüger P. The effect of keratinized mucosa width on peri-implant outcome under supportive postimplant therapy2015 January Epub.

9. Gabay E, Levin L, Zuabi O, Horwitz J. Plaque score change as an indicator for periimplant health in periodontal patients with immediately restored dental implants*Implant Dent* 2015; 24: 323–327.

10. Göthberg C, Gröndahl K, Omar O, Thomsen P, Slotte C. Bone and soft tissue outcomes, risk factors, and complications of implant-supported prostheses: 5-Years RCT with different abutment types and loading protocols*Clin Implant Dent Relat Res* 2018; 20: 313–321.

11. Grunder U, Polizzi G, Goené R, *et al.* A 3-year prospective multicenter follow-up report on the immediate and delayed-immediate placement of implants*Int J Oral Maxillofac Implants* 1999; 14: 210–216.

12. Karoussis IK, Müller S, Salvi GE, Heitz-Mayfield LJA, Brägger U, Lang NP. Association between periodontal and peri-implant conditions: a 10-year prospective study*Clin Oral Implants Res* 2004; 15: 1–7.

13. Máximo MB, de Mendonça AC, Alves JF, Cortelli SC, Peruzzo DC, Duarte PM. Peri-implant diseases may be associated with increased time loading and generalized periodontal bone loss: preliminary results*J Oral Implantol* 2008; 34: 268–273.

14. Mohanty R, Sudan PS, Dharamsi AM, Mokashi R, Misurya AL, Kaushal P. Risk Assessment in Long-term Survival Rates of Dental Implants: A Prospective Clinical Study*J Contemp Dent Pract* 2018; 19: 587–590.

15. Pjetursson BE, Helbling C, Weber H-P, *et al.* Peri-implantitis susceptibility as it relates to periodontal therapy and supportive care*Clin Oral Implants Res* 2012; 23: 888–894.

16. Polizzi G, Grunder U, Goené R, *et al.* Immediate and delayed implant placement into extraction sockets: a 5-year report*Clin Implant Dent Relat Res* 2000; 2: 93–99.

17. Rodrigo D, Martin C, Sanz M. Biological complications and peri-implant clinical and radiographic changes at immediately placed dental implants. A prospective 5-year cohort study*Clin Oral Implants Res* 2012; 23: 1224–1231.

18. Schropp L, Wenzel A, Stavropoulos A. Early, delayed, or late single implant placement: 10-year results from a randomized controlled clinical trial*Clin Oral Implants Res* 2014; 25: 1359–1365.

19. Uribarri A, Bilbao E, Marichalar-Mendia X, Martínez-Conde R, Aguirre JM, Verdugo F. Bone Remodeling around Implants Placed in Augmented Sinuses in Patients with and without History of Periodontitis*Clin Implant Dent Relat Res* 2017; 19: 268–279.

20. Wahlström M, Sagulin G-B, Jansson LE. Clinical follow-up of unilateral, fixed dental prosthesis on maxillary implants*Clin Oral Implants Res* 2010; 21: 1294–1300.

21. Zetterqvist L, Feldman S, Rotter B, *et al.* A prospective, multicenter, randomized-controlled 5-year study of hybrid and fully etched implants for the incidence of peri-implantitis*J Periodontol* 2010; 81: 493–501.
